# On the Development of an Acoustic Image Dataset for Unexploded Ordnance Classification Using Front-Looking Sonar and Transfer Learning Methods

**DOI:** 10.3390/s24185946

**Published:** 2024-09-13

**Authors:** Piotr Ściegienka, Marcin Blachnik

**Affiliations:** 1 Joint Doctoral School, Silesian University of Technology, 44-100 Gliwice, Poland; piotr.sciegienka@polsl.pl; 2 SR Robotics Sp. z o.o., Lwowska 38, 40-389 Katowice, Poland; 3 Department of Industrial Informatics, Silesian University of Technology, 44-100 Gliwice, Poland

**Keywords:** sonar data, front-looking sonar, deep learning, UXO, unexploded ordnance, AUV, underwater vehicle, transfer learning

## Abstract

This research aimed to develop a dataset of acoustic images recorded by a forward-looking sonar mounted on an underwater vehicle, enabling the classification of unexploded ordnances (UXOs) and objects other than unexploded ordnance (nonUXOs). The dataset was obtained using digital twin simulations performed in the Gazebo environment utilizing plugins developed within the DAVE project. It consists of 69,444 sample images of 512 × 399 resolution organized in two classes annotated as UXO and nonUXO. The obtained dataset was then evaluated by state-of-the-art image classification methods using off-the-shelf models and transfer learning techniques. The research included VGG16, ResNet34, ResNet50, ViT, RegNet, and Swin Transformer. Its goal was to define a base rate for the development of other specialized machine learning models. Neural network experiments comprised two stages—pre-training of only the final layers and pre-training of the entire network. The experiments revealed that to obtain high accuracy, it is required to pre-train the entire network, under which condition, all the models achieved comparable performance, reaching 98% balanced accuracy. Surprisingly, the highest accuracy was obtained by the VGG model.

## 1. Introduction

Underwater activities, such as laying cables or pipes and constructing underwater structures (e.g., seabed anchors for wind farms), require comprehensive knowledge about the locations where these objects are planned to be built. Among the various types of marine research that must be conducted, one of the key tasks is to check for the presence of hazardous objects lying on the seabed. These can pose potential dangers to working personnel or machinery. The importance of this issue was depicted in [1], the authors of which gathered a wealth of publicly available information on reported hazards related to sunken weapons, including physical contact, environmental contamination, and underwater explosions in the Baltic Sea and Skagerrak Strait areas. The sources of this problem were described by Bełdowski, Brenner, and Lehtonen in [2]. In their work, they presented the origin of the problem, which dates back to World War II and the post-war period when large-scale dumping of unused munition and chemical weapons occurred on the seabed of these bodies of water. Currently, due to corrosion, these objects pose a serious threat. For instance, in the specified dumping areas, it is estimated that there are about 50,000 and 190,000 tons of sunken munitions, respectively. The issue of identifying and retrieving these objects has been the focus of many recent international projects, such as MERCW [3], CHEMSEA [4], DAIMON [5], and MODUM [6].

Of course, not only European countries have issues with sunken hazardous objects. For example, the U.S. Department of Defense’s (DoD) two environmental research programs (the Strategic Environmental Research and Development Program (SERDP) and the Environmental Security Technology Certification Program (ESTCP)) are currently focused on developing systems for detecting, classifying, and locating dangerous unexploded ordnances (UXOs) submerged in water bodies. They are leveraging the experience gained from developing a similar system for land areas (it is estimated that about 10 million acres of US coastline and territorial waters may be contaminated with sunken munitions).

One of the main technological challenges is the selection of appropriate sensors and research platforms that account for the limitations of the aquatic environment. This issue was discussed by the authors of [7]. Among the available technologies and sensors for detecting UXOs, sonar systems are some of the most intensively used, allowing for effective scanning of the seabed, even in low-visibility conditions. This issue particularly affects the Baltic Sea area, where visibility is very low, making the use of visual images very limited.

In [8], various approaches to automated target recognition (ATR) were discussed—in particular, the automation of the detection and classification of underwater mines. This problem is typically addressed using different machine learning methods, including classical feature extraction methods such as SIFT [9], convolutional neural networks (CNNs), and transfer learning [10]. However, these methods require large amounts of training data, which are difficult to obtain. Additionally, the collected data should match the characteristics of the system that will be used for the classification of hazardous objects. This includes the type of sonar (such as Synthetic Aperture Sonar (SAS), Side-Scan Sonar (SSS), or Forward-Looking Sonar (FLS)), the system installation location (stationary, remotely operated vehicle (ROV) or autonomous underwater vehicle (AUV)), its frequency characteristics, and the height at which it will operate.

Among the available datasets used to build machine learning systems for the classification of UXO/nonUXOs, the TREX dataset [11] is commonly utilized. This dataset was developed as part of research conducted by the Applied Physics Laboratory at the University of Washington in the Gulf of Mexico region. It includes 33 objects scanned over a full 360° aspect range; a 5–30 kHz frequency band; and deployment ranges of 5–40 m, corresponding to grazing angles from 36° to 5°, respectively.

However, in the practical scanning of water bodies, AUV-type vehicles are commonly used, on which relatively inexpensive FLS can be installed. The operating characteristics of such FLS differ significantly from those of SAS, leading to difficulties in accessing suitable training data for the building UXO/nonUXO classifiers. To address these challenges, in our research, we focused on preparing a dataset designed for the classification of UXO objects using FLS. The study assumed that an AUV-class vehicle would operate at heights ranging from 1 to 3 m above the seabed, recording data using multibeam imaging sonar operating at frequencies from 900 kHz to 3 MHz.

Due to the difficulty in accessing real-world data, the dataset was developed using a simulation tool described in [12]. During simulation preparation, it was assumed that UXO objects would correspond to typical hazardous ammunition with calibers in the range of 80 mm to 200 mm, while nonUXO objects included all other objects—in particular, objects of pipe-like or box-like shapes that are too large or too small to be considered UXOs. Additionally, since the FLS sensor is typically mounted on a moving platform, each object was recorded three times at the top of the acoustic image (when the vehicle first approaches the object), in the center of the image, and in the bottom of the image right before leaving the view area of the sonar (i.e., when closest to the object).

In the next step of the research, the created dataset comprising 69,444 images was used to assess state-of-the-art machine learning models using the transfer learning approach. In this context, various types of neural networks were examined, including VGG16 [13], different ResNET [14] models, ViT [15], RegNet [16], and Swin Transformer [17]. This research aimed to define a reference performance for the off-the-shelf models, which can be used for comparison with other models designed for this dataset.

This article introduces the following two novelties:

- We introduce a dataset designed for UXO/nonUXO classification using acoustic images recorded using a digital twin of front-looking sonar.
- We define a reference performance for UXO/nonUXO classification using standard, off-the-shelf models applying transfer learning methods.

This article is structured as follows. Section 2 describes related work in terms of machine learning applications to sonar images, as well as synthetic data generation. Section 3 describes how the dataset was generated, including all of the details of image generation. Next, in Section 4, neural network architectures that were used in the experiments are described. After that, Section 5 describes the obtained classification results. Section 6 concludes the the article, suggesting directions for future research.

## 2. Related Work

To search for and identify sunken objects, including UXOs, many different sensors can be used. Among the most popular are magnetometers [18,19,20] and sonar [21]. Sonar allows for the observation of the bottom surface of water bodies by analyzing the acoustic wave emitted by the device, reflected from an obstacle, then registered by the detector. Although the data obtained in this way are characterized by relatively low resolution and are susceptible to various types of interference, they enable the automatic detection of objects in the underwater environment [22,23,24].

Machine learning methods are probably the most popular tool used for the analysis of sonar data. The authors of [25] discussed various aspects of their use, particularly emphasizing deep convolutional neural networks (CNNs). They also addressed issues related to the quality of sonar images obtained for analysis, which are affected by phenomena such as non-homogeneous resolution, non-uniform intensity, speckle noise, acoustic shadowing, acoustic reverberation, and multipath problems. They also discussed the issue of the limited amount of sonar data available for the training of models and the use of transfer learning methods to fine tune models pre-trained in similar domains for the domain of sonar images using a relatively small dataset.

The concept of using pre-trained models is frequently employed by various researchers. Two main approaches are applicable here. In the first one, pre-trained neural networks are utilized to extract significant features from images, thereby obtaining a simplified numerical representation of the image. Subsequently, various predictive models, such as SVM, are constructed within this feature space [26]. The second approach is based on fine tuning of the model [27].

In the field of image processing, classification and detection are two distinct tasks. Detection is a somewhat more complex problem, as it requires specifying the location of the object. A classical tool in this domain is the YOLO neural network [28]. The authors of [29] used this network in combination with the ShuffleNetv2 network. In addition, the authors employed similarity features for migration learning, enhancing model training and enabling improved detection performance.

In the classification of UXO/nonUXO objects, numerous studies have been based on the TREX dataset. For example, the authors of [30] demonstrated the application of various deep neural network concepts. In contrast, the authors of [31] employed three-dimensional convolutional neural networks for SAS data. A slightly different approach in the field of munition detection utilizing sequence models was proposed in [32]. The main idea of this approach was the efficient modeling of the spatially correlated nature of the scattering features.

Many researchers have highlighted the issue of insufficient training data ti build classifiers and detectors using machine learning methods. A common solution in this case is the generation of synthetic data. Sonar image generation methods can be divided into several groups. Those in the first group rely on the use of machine learning methods, while others rely on computer simulation methods such as the finite element method (FEM) or ray tracing.

Within the first group, applications of neural networks such as generative adversarial networks (GANs) can be found. An example of this approach was reported in [33]. In [34], the authors applied the style transfer method to generated 3D objects. The proposed solution takes a single channel-depth image from a simulator to provide various aspects (e.g., orientation, scale, and translation) of the captured data.

The mentioned approach based on the physical modeling of phenomena corresponding to sonar acoustic imaging is described in [12]. The proposed solution is a complete virtual environment where the operation of a multibeam sonar is simulated, allowing for the placement of various objects and the subsequent receipt of signals recorded by the virtual device. A sonar simulator reflecting the operation and physical aspects of the environment was also developed in [35]. The application of Finite Element Method (FEM) modeling to SAS was described, among other methods, in [36], where the comparative results of which indicate an almost perfect representation of scanned objects by their digital twins. A similar method was used in the development of the TREX dataset [11], which is one of the most frequently used datasets for UXO/nonUXO object recognition. In this dataset, alongside real scans, some records are supplemented with their digital twins. However, this dataset was created for classification problems obtained for SAS sonar acoustic images, so it does not meet the needs of the research conducted in this article.

## 3. Dataset Generation

Machine learning methods, especially deep networks, require very large amounts of training data. In the case of the analysis and processing of standard images depicting various objects, there are many publicly available datasets, such as ImageNet or COCO, which are very popular. However, in the case of sonar data, the situation is more complicated. Signals recorded from sonar can be in raw form (a table with information on the intensity of the signal reflected from an obstacle, coming from a given direction and distance) or processed into a standard graphic image using the selected transforming function. Sonar data can come from different types of devices, such as side-scan sonar or front-looking sonar, where the final representation of the data differs between the two cases because the purpose of these devices is different. Side-scan sonar is used to map large areas of the seafloor from greater heights above the seabed. Front-looking sonar, on the other hand, is used to scan the bottom directly in front of a moving vehicle; most often, it operates at higher frequencies than side-scan sonar. Another important aspect when collecting data is the operating parameters of the device—both internal (resolution, viewing angle, and range) and external (orientation of the sonar mounted on the measurement platform and height above the bottom). The above factors, as well as the fact that collecting data in underwater environments is extremely expensive and time-consuming, result in very few publicly available sonar datasets that contain relatively small numbers of samples.

This problem is even more important for UXO classification problems, where, as already indicated, no dataset is available for FLS. Below, we discuss the process of the development of the UXO/nonUXO classification dataset, starting from the problem of determining the angle of FLS observation and preserving the resolution and height limitations. Additionally, we describe all other constraints applied when designing the dataset.

### 3.1. The Virtual Environment Used in Simulations

**Figure 1:**
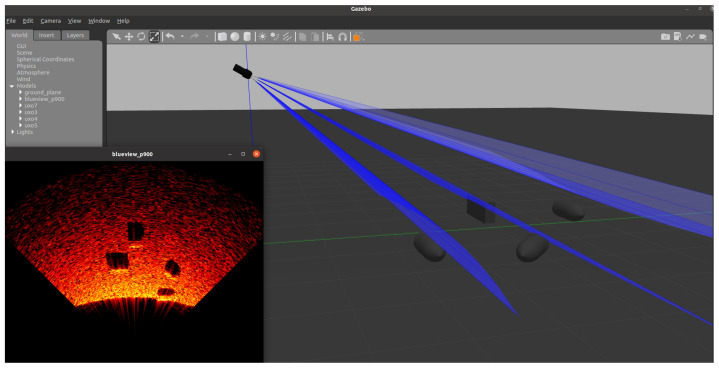
Generator—view of the simulation environment.

Due to the lack of sonar data, when working with machine learning (ML) methods, it is common practice to prepare training sets by generating them [27]. This approach was also used in this work. In particular, we used a robot simulation software called Gazebo v.11.13.0 (https://gazebosim.org/home), which enables realistic modeling of 3D environments, along with an additional software package called ‘Project DAVE’ v4.3.0 intended for the simulation of underwater environments. Project DAVE offers advanced sensor models such as front-looking sonar, among other advantages [12]. Based on the above modules, an original solution was created, enabling the generation of 3D models of the examined objects with predefined dimensions, placing them in a virtual environment and a specific position and orientation, then simulating the passage of a virtual sonar over the object. Additionally, the sonar was simulated at a given height above the bottom (see Figure 1). The sonar output is in PNG image format, presenting the area seen by the sonar on a section of the graph in the polar coordinate system. The entire process was automated, allowing for the generation of tens of thousands of sonar images based on defined case descriptions.

**Figure 2:**
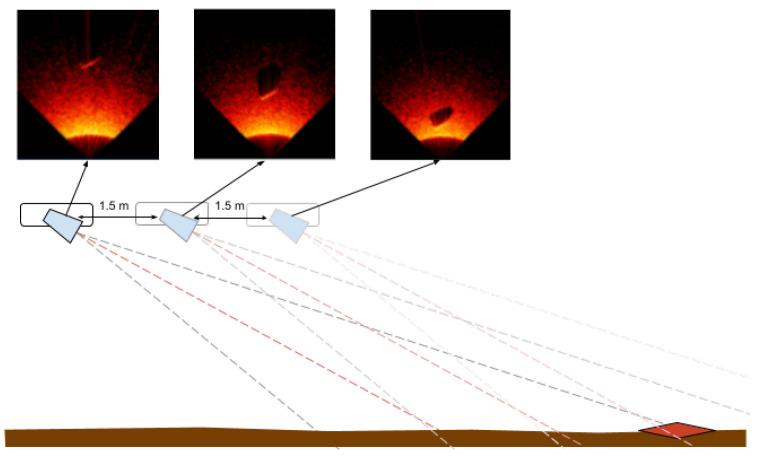
Generator—principle of operation.

**Figure 3:**
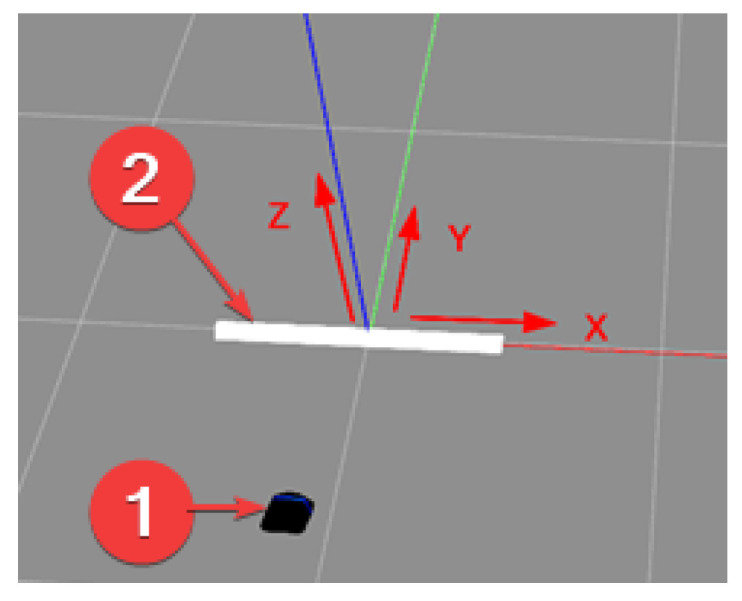
Generator—coordinate system. 1—sonar; 2—test object.

The authors prepared a comprehensive solution including the preparation of the tested objects, selection of parameters of the data generation process, and automatic generation of the entire set. For that purpose, a Python script was developed that allows for the placement and management of objects in the virtual space and simulation of the process of moving virtual sonar over an object. Additionally, the data recording system was designed to allow for the generation of a scene based on the configuration file, including the randomness of specific scene and object parameters. Configuration parameters for the sonar include virtual sonar parameters such as the number of beams, frequency, horizontal and vertical viewing angles, and minimum and maximum range, as well as the shape of the sonar’s path over the object. By default, the sonar moves straight ahead at a height of 1–3 m above the seabed directly above the object. Data are recorded at the following three distances between the sonar and the object: 3 m, 1.5 m, and 0 m. This simulates the process of passing over the tested object. In a real environment, sonar data are recorded continuously (e.g., 15 fps @1.5 m/s), while the classification module receives the following three consecutive recorded images with a specified frequency: at the present location and recorded 1.5 m and 3 m from the object. The distance of 1.5 m between the images was selected based on an analysis that took into account the range of the sonar and the other developed parameters of the measuring platform so that the ’photographed’ object was at the bottom, in the middle, and at the top of the received ’photograph’. In this work, each of these three images was classified independently. But the generated dataset allows for the consideration not only of a single image as input to a classifier but also a set of three frames/images that may further improve classification performance. The discussed data generation method is presented in Figure 2 and the coordinate system used is presented in Figure 3.

In the developed system, the description of the object is loaded from SDF (Simulation Description Format) [37] file. The 3D model of the object can be obtained by any 3D graphics program. The generator can work in two modes; it can generate data based on prepared models of 3D objects, which are then subjected to random transformations, changing their size and orientation within specific, configurable limits, or it can generate data based on precisely defined parameters read from the configuration file for each generated case. When preparing the data for the purpose of this work, the generator operated in the second mode, creating data compatible with the magnetometric dataset described in [38].

To make the simulated objects of the UXO and nonUXO classes compatible with the magnetometric dataset, its parameters and shapes were determined accordingly. However, in the developed dataset not all nonUXO objects with available magnetometric data have corresponding objects in the sonar data. Some objects were ignored. The parameters and shapes of the UXO objects are outlined as follows:

- Cylindrical/pipe-shaped objects diameters (corresponding to the caliber of the projectile) in the range of 80 mm to 200 mm and lengths ranging from 350 mm to 650 mm.

The respective nonUXO objects are defined as follows:

- Objects too small to be dangerous (marked as tooSmall), usually objects resembling UXOs but with a slightly smaller diameter, i.e., 45 mm to 70 mm in diameter and 150 mm to 300 mm in length;
- UXO-shaped objects that are too large (marked as tooBig), i.e., pipe-type objects with the following parameters: diameter of 250 mm to 400 mm and length of 750 mm to 1200 mm;
- Box-shaped objects with lengths of 900 mm to 2000 mm, widths of 50 mm to 200 mm, and thicknesses of 50 mm to 200 mm.

**Figure 4:**
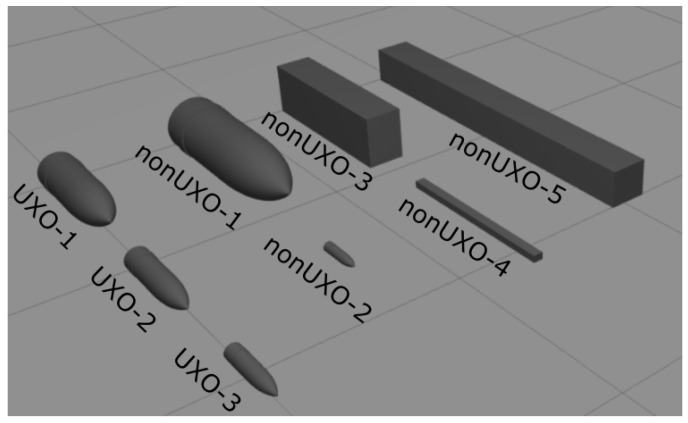
Sample 3D models for UXO- and nonUXO-class objects.

The shapes of the UXO and nonUXO objects were developed to surround the class of UXO objects. Therefore, the tooSmall and tooBig classes surround real UXO objects. The box-shaped class was also considered similar to UXOs but with different elongation parameters—being too long to be considered a UXO (in practice, this may represent rail-like objects with similar diameters but longer lengths). A visualization of the example shapes is shown in Figure 4.

The results of the generator’s operation are available at https://www.kaggle.com/datasets/piotres/front-looking-sonar-uxo (accessed on 30 June 2024).

Here, a single tested case is represented by three graphic files in PNG format, containing the view recorded by the sonar presented in the form of a section of a graph in the polar coordinate system. The file name consists of information separated by an underscore character, specifying the following parameters used during image generation: [1]_S[2]_OD[3]_[4]_[5]_OP[6]_[7]_[8]_OO[9]_[10]_[11]_[12], where

1. Object class: UXO/nonUXO;
2. Sonar height above the bottom [m];
3. Object size on the X axis [m];
4. Object size on the Y axis [m];
5. Object size on the Z axis [m];
6. Position of the object on the X axis [m];
7. Position of the object on the Y axis [m];
8. Position of the object on the Z axis [m];
9. Object rotation around the Y axis [deg];
10. Object rotation around the Z axis [deg]
11. Name of the 3D model used;
12. Flow step index: from 1 to 3.

The following assumptions were made for the generation of sonar data for the purposes of this work:

- Sonar angle towards the bottom on the measurement platform: 30°;
- Height of the measuring platform above the bottom: random value in range 1 to 3 m;
- 3 images recorded for a single case (object at the end, in the middle, and at the beginning of the sonar visibility range) (see Figure 2);
- ‘Burying’ of the object in the bottom to a maximum of half its height (determined by random generation).

**Figure 5:**
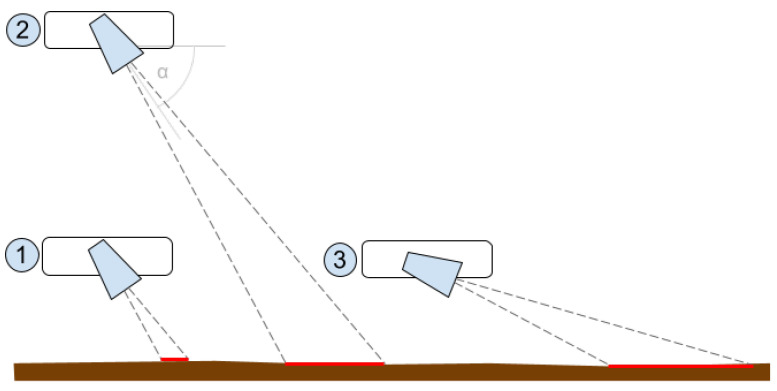
Analysis of sonar angle and bottom visibility range.

**Figure 6:**
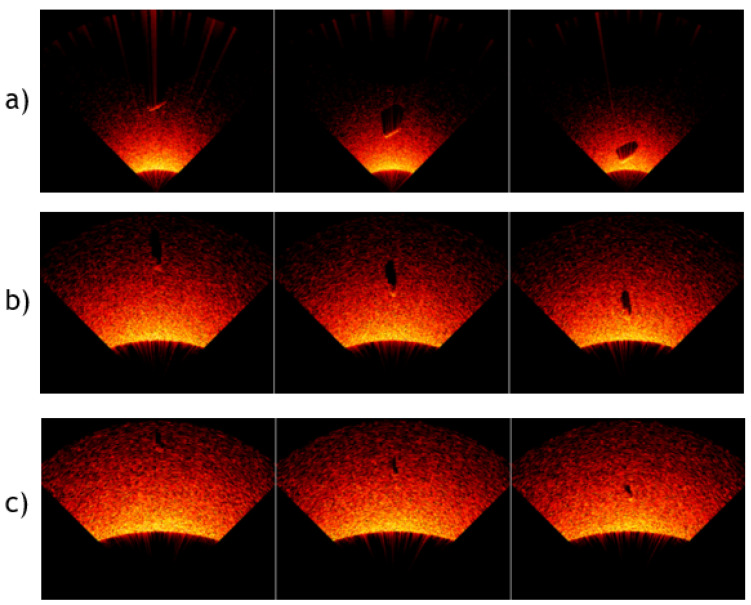
Sample of generated sonar images. (**a**) Sonar height: 1.03 m; object dimensions: L = 1.03, Ø = 0.31 m. (**b**) Sonar height: 2.30 m; object dimensions: L = 0.95, Ø = 0.39 m. (**c**) Sonar height: 2.95 m; object dimensions: L = 0.77, Ø = 0.27 m.

The sonar angle of 30° was selected on the basis of an analysis taking into account the parameters of the sonar used in the research, the dimensions of the object, and the target application in which the developed solution is to be used. The sonar resolution, vertical viewing angle, range, and angle of inclination relative to the bottom are the basic parameters affecting the bottom visibility range. Figure 5 shows the influence of the sonar height above the bottom and the sonar inclination angle on the bottom visibility range; the lower the height and the greater the inclination angle (1), the smaller the field of view and the greater the height (2) and/or the smaller the tilt angle (3), the larger the field of view. Due to the fact that a magnetometer is placed on the measurement platform, together with the sonar, the height of the platform above the bottom was determined to be 1–3 m. This is the optimal value for the detection of magnetic objects, since, on the one hand, the system is supposed to detect relatively small objects with a minimum size of 45 mm, while, on the other hand, when the sonar is located close to the bottom (1 m), when viewing the largest objects (2000 mm), the angle should be smaller to fit the object into the visibility range. Performed calculations in the virtual environment confirmed that the optimal inclination angle is 30°. Example sonar images for three different objects at different heights are presented in Figure 6; the three pictures in a row show the steps representing the process of flowing the measurement platform over the object.

A summary of the parameters of the simulation environment, except the UXO/nonUXO object details, are presented in Table 1.

### 3.2. The Dataset Characteristics

Using the virtual environment described in the previous section, 69,444 sonar images were generated for 23,148 different cases. Among them, 30% belong to the UXO class and 70% to the negative class of nonUXO objects.

This dataset was then divided into three parts, allowing for machine learning model evaluation. The split was made twice—once using a simple training, validation, and testing split and the second time to allow for the performance the five-fold cross-validation procedure. In the first case, the proportion of the number of cases in each subset was as follows: training, 65%; validation, 5%; testing, 30%. For the second case, the split was 72%/8%/20%, that is, 20% of the cases were allocated to the test set. Of the remaining 80% of the training data, 10% was designated as validation data. The 10% from the training set corresponds to 8% of the total size of the dataset. The detailed statistics are presented in Table 2.

It is worth paying attention to the fact that when dividing data into individual sets, all three sonar images of the same object are always located only in one of the sets. This is to protect against the information leak effect and to avoid overestimation of results.

The final dataset including the split for cross validation and for the training, validation, and test sets is available at https://www.kaggle.com/datasets/piotres/front-looking-sonar-uxo (accessed on 30 June 2024).

## 4. Prediction Model Evaluation

Due to the development of image classification methods, the most common approach when dealing with new image classification tasks is to apply transfer learning methods. This approach allows for the utilization of pre-trained models developed using large computing resources and massive image datasets as the basis of image feature extraction and as a good initialization step in new tasks. In this article, we followed this path, comparing the most commonly used pre-trained models for UXO/nonUXO classification. In the experiments, we evaluated the most commonly used and the most popular network architectures, including VGG16, two ResNet models (ResNet34 and ResNet50), RegNet, Visual Image Transformer (ViT), and Swin Transformer.

All of the evaluated models were initially pre-trained using the 1M ImageNet dataset, which contains images of real-word objects that are significantly different from sonar images. Therefore, the models were evaluated in two stages—by adapting just the last fully connected layers or pre-training the entire network. For ResNet, which is the most widely used image classification architecture, we also evaluated various depths of training, adding additional network blocks to the training process. This allowed us to verify how deeply the network needs to be trained to achieve the desired accuracy.

### 4.1. Model Architectures

Neural network architectures have significantly evolved. Below, we present a short overview of the models we used in the experiments.

#### 4.1.1. VGG Architecture

VGG16 is a relatively old, standard convolutional deep neural network developed by the Visual Geometry Group (VGG) at the University of Oxford. This model was presented in the article “Very Deep Convolutional Networks for Large-Scale Image Recognition” [13] and gained popularity after achieving very good results in the ImageNet 2014 competition. VGG16 is known for its architectural simplicity and effectiveness in image classification tasks.

**Figure 7:**
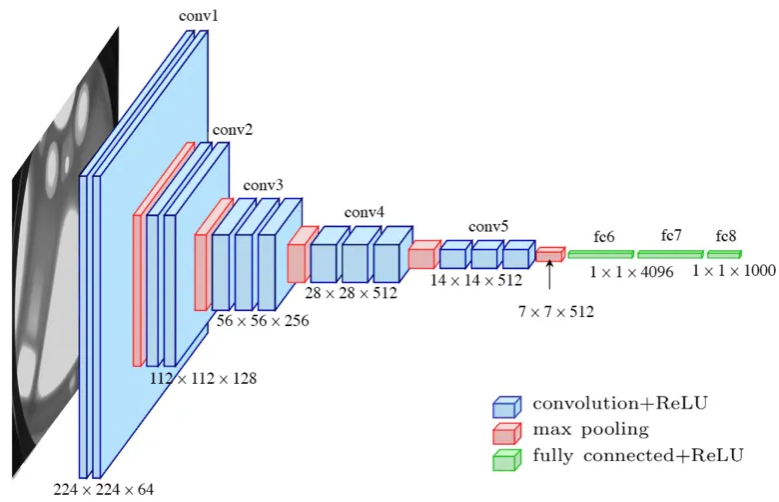
VGG-16 model architecture [39].

VGG16 consists of 16 convolutional and fully connected layers, which gives it its name. All convolutional layers in VGG16 use small 3 × 3 filters followed a 2 × 2 max pooling layer. The max pooling layer reduces the size of an output tensor, and the number of filters in each layer doubles to maintain the important information. The architecture of the VGG16 model is shown in Figure 7.

The disadvantages of this model include high demand for computational resources and long training time due to its depth, number of parameters, and lack of skip connections, which intensifies the problems of a vanishing gradient and a large number of parameters. This is especially problematic when training a model from s7 cratch.

#### 4.1.2. ResNet Architecture

**Figure 8:**
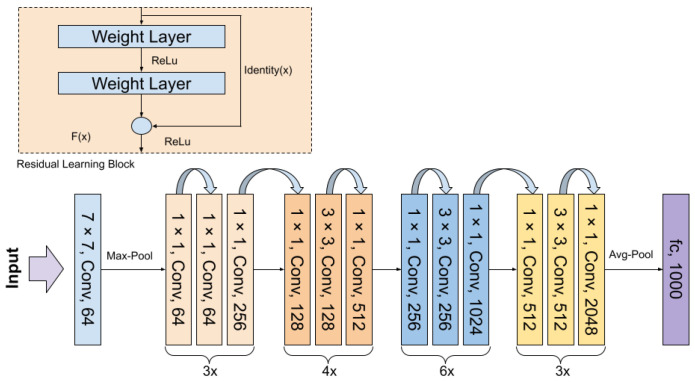
ResNet model architecture [14].

Residual Networks (ResNets) are a family of architectures that introduce an innovative approach to training very deep neural networks using residual blocks and so-called skip connections, preventing the vanishing gradient problem. Residual blocks consists of convolutional layers, typically with a ReLU activation function, followed by batch normalization. This architecture has had a significant impact on the field of computer vision and is widely used in various image processing tasks [14]. In the experiments, two variants of the ResNet architecture were used, namely ResNet34 and ResNet50. The latter has a larger number of layers, utilizing the bottleneck blocks, which consist of three layers (1 × 1, 3 × 3, and 1 × 1 convolutions) instead of the two-layer blocks used in ResNet34. The general architecture is shown in Figure 8.

#### 4.1.3. RegNet Architecture

RegNet, or Regular Network, is a family of deep learning architectures developed by Facebook AI [16]. RegNet architectures share several properties; they are not manually designed like VGG or ResNet but are developed by exploring the design space using an AnyNetX method. This allows the user to specify the relation between simplicity, scalability, and effectiveness of the network. Such an architecture is organized similarly to ResNet into a block structure, where each block is made up of convolutional layers, batch normalization, and ReLU activation.

The disadvantage of this model is that it is task-specific, since its structure is designed for a specific goal. Many pre-trained RegNet models are available in repositories. In our experiments, the RegNetY800MF model was used with additional Squeeze-and-Excitation (SE) blocks that recalibrate channel-wise features, slightly improving its accuracy.

#### 4.1.4. ViT Architecture

Vision Transformer (ViT) is an innovative model in computer vision that introduces techniques used in natural language processing (NLP) models for image analysis. It was proposed in “An Image is Worth 16 × 16 Words: Transformers for Image Recognition at Scale” [40] by the Google research team in 2020. ViT shows that transformer models, which have achieved breakthrough results in NLP, can also be effectively applied to image processing tasks.

**Figure 9:**
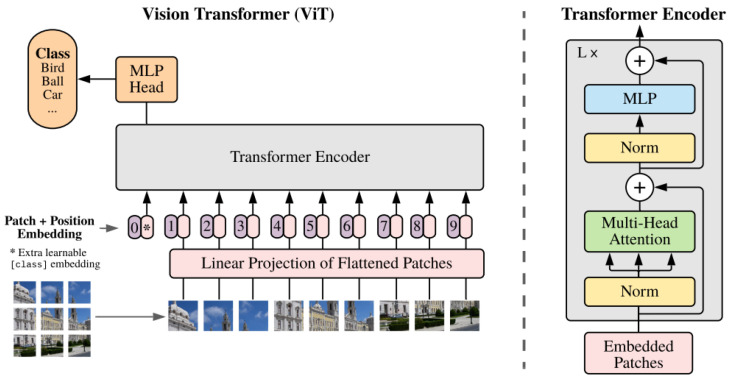
ViT model architecture [40].

The basic idea of ViT is to transform an image into a sequence of “words”, which are then processed by a standard transformer architecture as shown in Figure 9.

The main advantage of ViT is its exceptional scalability, especially for large datasets such as JFT-300M or ImageNet-21k, where it often outperforms traditional convolutional networks.

However, ViT has its limitations. It requires large datasets to train effectively, which poses a challenge in cases where such data are not available. Additionally, ViT training and inference require significant computational resources, including large amounts of memory and processing power, which may increase the costs of implementing and maintaining the model in production.

#### 4.1.5. Swin Transformer Architecture

The Swin Transformer (Shifted Window Transformer) [17] is a type of vision transformer designed to efficiently handle high-resolution images for tasks such as image classification, object detection, and semantic segmentation. It introduces a hierarchical architecture that processes images at multiple scales, similar to convolutional neural networks (CNNs). The key innovation in Swin Transformer is the use of “shifted windows”, where non-overlapping windows are shifted across different layers to allow for cross-window connections, improving the model’s ability to capture global and local information. This design leads to improved computational efficiency and scalability while maintaining strong performance on various vision tasks.

**Figure 10:**
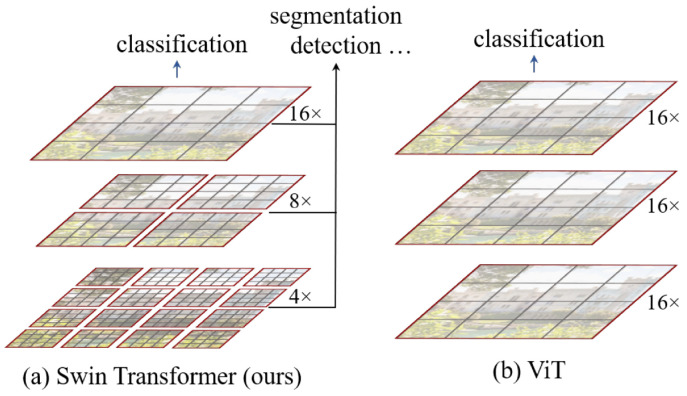
The concept of hierarchical feature maps and merging image patches of Swin Transformer (**a**) vs. the approach used in ViT (**b**) [17].

The difference between Swin Transformer and ViT is shown in Figure 10, where the idea of hierarchical feature maps with merging image patches is presented.

### 4.2. Model Evaluation Procedure

First, in each model, the final layer was modified to ensure the execution of a binary classification task, i.e., UXO/nonUXO, so that the output layer consisted of two neurons. The number of fully connected hidden layers were always set to two. Next, an early stopping mechanism was implemented, interrupting the training process if there was no improvement in the value of the loss function by at least min_delta=0.0005 (determined experimentally) for 10 consecutive epochs. The Adam (Adaptive Moment Estimation) optimizer was used to train the model. In the experiments, we encountered a problem with convergence of the models; therefore, various learning rates were evaluated in order to find the learning rate that assures convergence of the model. The loss function for all models was the cross-entropy loss. In the experiments, batch_size=32.

**Figure 11:**
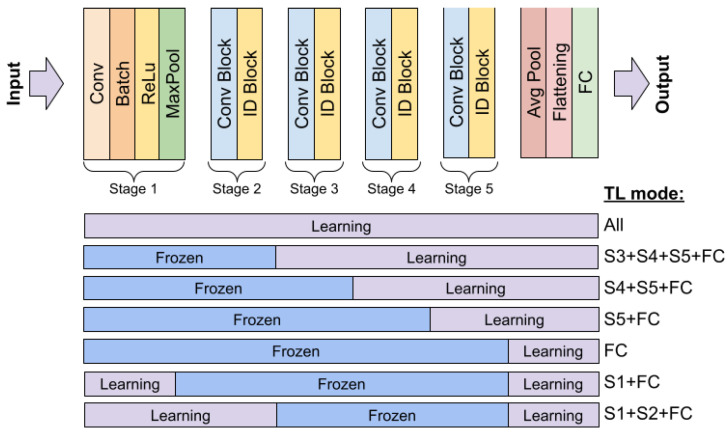
Diagram showing the freezing of individual layers of the ResNet model.

The initial evaluation and parameter selection were conducted using a train–test split procedure to reduce computation time. In addition, since transfer learning allows for the retraining of the models with different depths, we used ResNet50 to assess the influence of the depth of the model on the final performance. Figure 11 shows the evaluated ResNet blocks.

Based on the obtained results, the remaining models were only evaluated by retraining the entire network.

**Figure 12:**
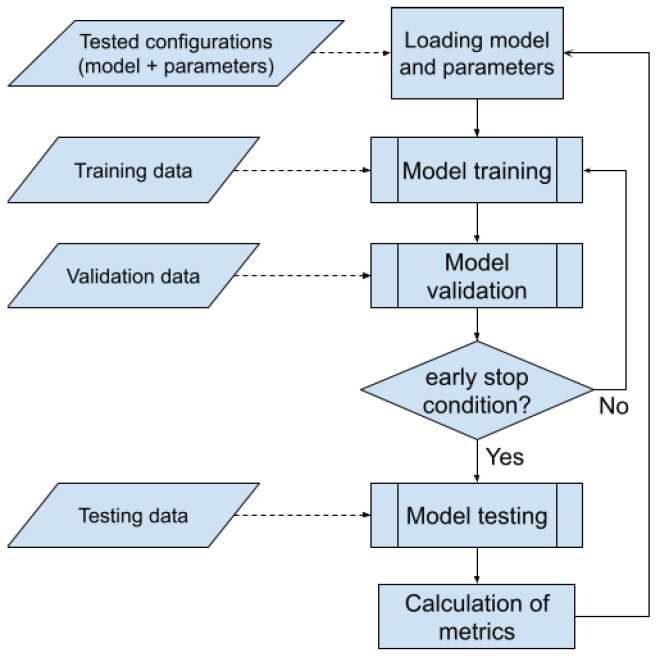
Diagram of the model training procedure.

The final quality of the model for the best set of parameters was assessed using a cross-validation procedure. The fine-tuning process of the models was carried out according to the procedure presented in Figure 12.

The experiments were performed using the PyTorch library and the torchvision module. The pre-trained models were obtained from the set of torchvision models and run on a server equipped with 64 logical cores and an NVidia Tesla T4 graphic card.

### 4.3. Metrics

To provide a comprehensive view of prediction quality that encompasses different aspects of the model’s properties, in our research, we used four different prediction performance metrics, namely average precision, balanced accuracy, Matthews correlation coefficient, and F1 score. All these metrics are designed for imbalanced data, and each provides a unique perspective on the model’s performance.

The average precision balances precision and recall and is important in cases where misclassifications can have serious consequences. Balanced accuracy ensures that the model is equally effective in identifying both classes. The Matthews correlation coefficient provides an overall view of the model’s performance, taking into account all aspects of the confusion matrix, while the F1 score is one of the most popular matrices used in unbalanced that measures the harmonic mean of the precision and recall.

## 5. Results

The experimental procedure consisted of several steps. First, we analyzed the process of the influence of the depth of the retraining procedure based on the ResNet50 architecture. Finally, based on the obtained results, the cross-validation procedure was used to assess the final performance. Herein, we present the silence maps obtained for the models, which allow us to identify the properties of the acquired knowledge. Below, we present the consecutive stages.

### 5.1. The Influence of Depth in Retraining the ResNet50 Model

**Figure 13:**
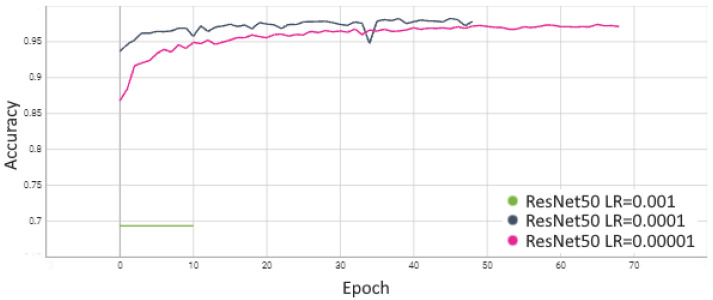
Comparison of accuracy for ResNet50 model.

**Figure 14:**
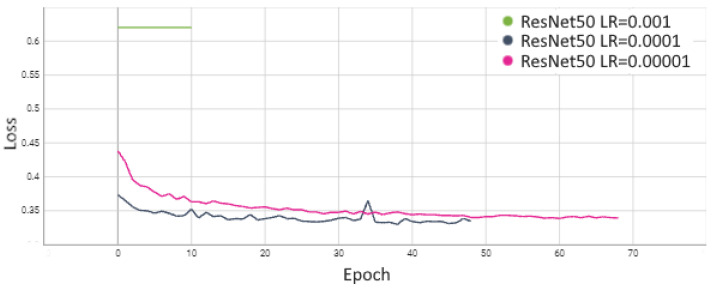
Comparison of loss function for ResNet50 model.

The process of ResNet50 evaluation started with an analysis of the learning rate (LR) selection. Studies were conducted for a scenario where Stage 5 and FC layers were fine-tuned. The obtained performances indicate that the best results were achieved with a learning rate of 0.0001. In contrast, increasing it to 0.001 caused a lack of convergence, and for a very small learning rate of LR = 0.00001, the network converged much more slowly, resulting in an accuracy that was 0.03 worse for AP and 0.022 for MCC. Based on these studies, optimization of the ResNet family of networks with a learning rate of 0.0001 was adopted for subsequent experiments. The detailed results are presented in Table 3, and a visualization of the training process is shown in Figure 13 and Figure 14.

The analysis of the impact of fine tuning groups of layers showed that the highest prediction performance was achieved by fine tuning all layers, while the worst results occurred when only the FC layers were fine-tuned. This difference was over 30 percentage points for AP, 0.3 for MCC, and about 18 percentage points for balanced accuracy. The second worst result was achieved with the fine tuning of Stage S1 and FC and Stage S5. However, in the latter case, the results were significantly better than for Stage S1 + FC by about 10 percentage points.

Fine tuning a larger number of convolutional layers, along with the FC, resulted in very good results, with outcomes close to those achieved with fine tuning of the entire network. Therefore, the key to achieving correct results is fine tuning several convolutional layers simultaneously. However, considering the properties of the learning process, including the number of adapted parameters, it is advisable to fine tune the entire network.

### 5.2. Comparison of Model Performance

The final comparison of the models was performed using five-fold cross-validation. The obtained performances is shown in Table 4.

The best results were obtained for the VGG16 model, although they were not statistically significantly different from the results obtained by the ResNet34 and RegNet800 models. For the statistical evaluation, a paired Student’s T test was used. However, there was a significant difference for the ResNet50 model and Swin-Transformer compared to VGG16. These results may stem from the properties of the networks, i.e., the VGG16 network has the proportionally most learning parameters, specifically 138,357,544, compared to 21,797,672 parameters for the ResNet34 network and 25,557,032 for the ResNet50 network. This high number of parameters may result in higher resistance for the noise present in sonar images. Unfortunately, this aspect requires additional research that is beyond the scope of this article.

Surprisingly poor results were obtained for the transformer-based models. For both ViT and Swin-Transformer, the results were significantly poorer than the results obtained for the VGG network, and the ViT transformer did not converge. This may be because image patching may not be suitable for very noisy images present in acoustic data. The shape of the objects may also influence the performance of ViT, where a single UXO often falls into a single patch, not allowing the model to identify the true class and to apply the benefits of the transformer’s architecture. In Swin Transformer, due to the hierarchical structure, the model can find relations between patches, allowing it to achieve reasonable performance, although still worse than that of the VGG network.

**Figure 15:**
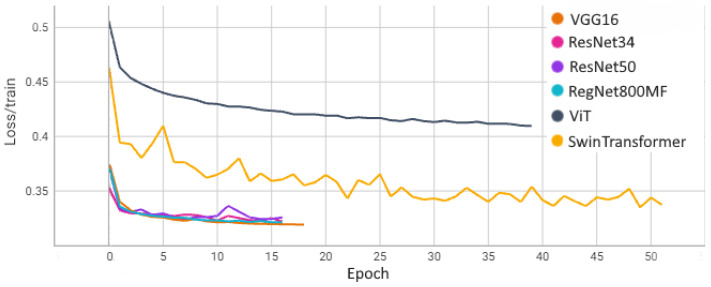
Learning curves on the training set.

**Figure 16:**
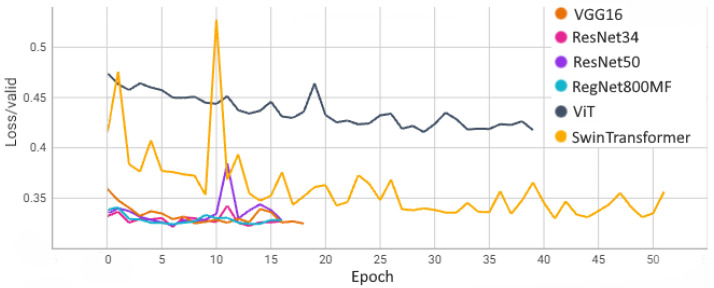
Learning curves on the validation set.

Figure 15 and Figure 16 show the learning curves for each model obtained on the training and validation set. These results were obtained on the train–test split without a cross-validation procedure.

### 5.3. Saliency Map Analysis

**Figure 17:**
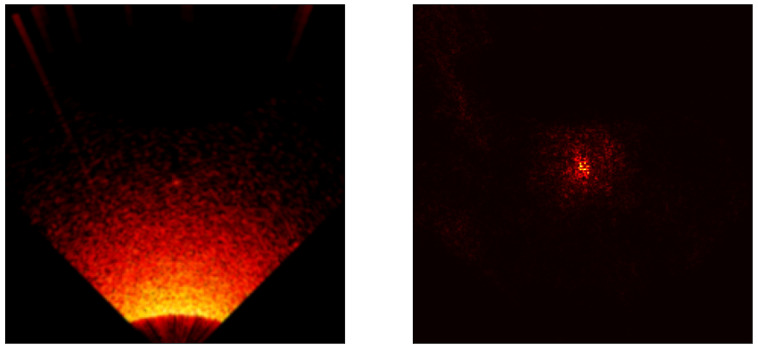
Example saliency map for the VGG model.

**Figure 18:**
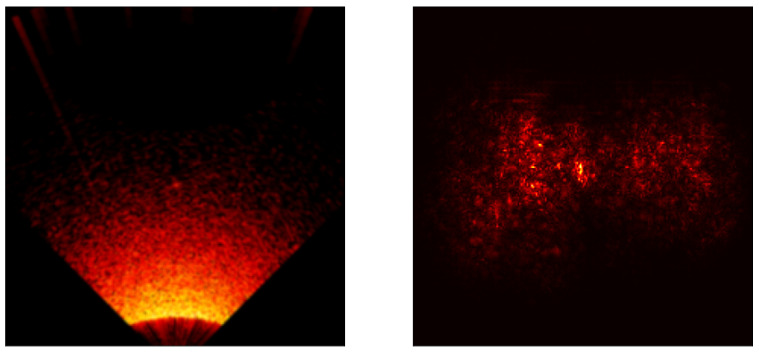
Example saliency map for the ResNet34 model.

**Figure 19:**
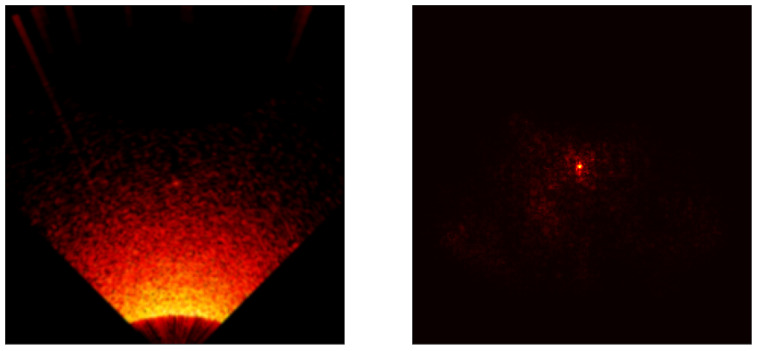
Example saliency map for the ResNet50 model.

**Figure 20:**
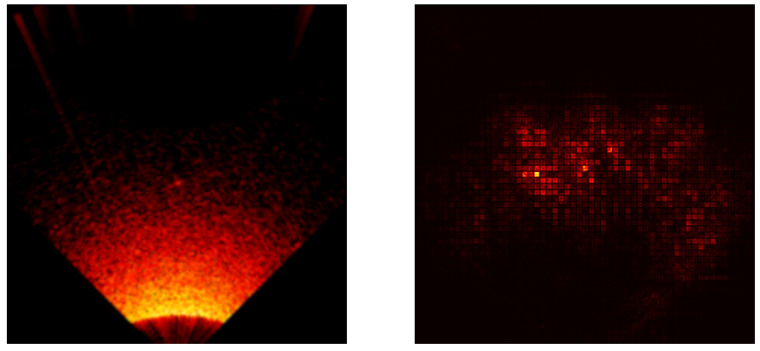
Example saliency map for the RegNet800 model.

**Figure 21:**
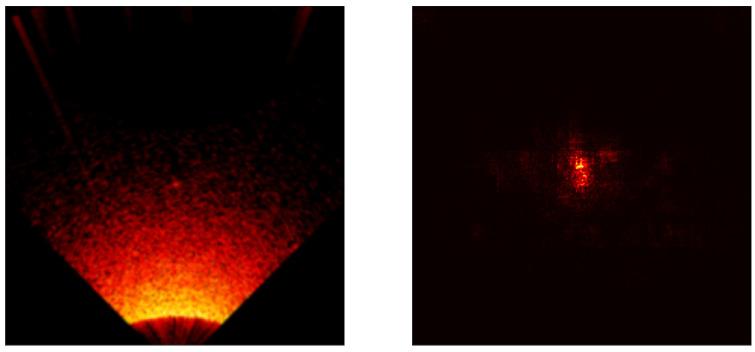
Example saliency map for the Swin Transformer model.

To extend the conducted research, each model was analyzed using the saliency map technique [41] to assess its properties. Saliency maps are visualizations used in machine learning, particularly in deep learning models, to highlight which parts of an input (an image) are most influential in making a prediction. Below are the results for all models included in the cross-validation process, except the ViT transformer, Figure 17, Figure 18, Figure 19, Figure 20 and Figure 21. As can be observed, for the sample image containing a UXO-class object, the models correctly identified the location where the classified object was present.

## 6. Conclusions

The aim of this research was to develop a dataset for UXO classification based on acoustic images from FLS and its evaluation using state-of-the-art, off-the-shelf neural network models. The conducted research allowed us to prepare such a dataset using a digital twin approach and objects augmented in the virtual environment utilizing open-sourced Gazebo software and plugins developed within the DAVE project, delivering tools for sonar simulations. When constructing the dataset, the UXO class representing munition-like objects with lengths of 350 mm to 650 mm and calibers of 80 mm to 200 mm was bounded by the nonUXO class, which contained objects smaller and larger than UXOs. In total, 69,444 sonar images were generated. The constructed environment allowed for the simulation of underwater vehicle movements such that the images were recorded in a group of three consecutive images of the same object. Next, this dataset was used to evaluate the state-of-the-art neural network architectures.

Tests were conducted on six different convolutional neural networks under evaluation, namely: VGG16, ResNet34, ResNet50, RegNet, ViT, and Swin Transformer. The obtained results indicated that among the evaluated networks, the VGG16 network outperformed other models, achieving F1=0.994 and MCC=0.987. The second place was occupied by RegNet (F1=0.994 and MCC=0.983), and ResNet34 (F1=0.991 and MCC=0.983) and ResNet50 (F1=0.990 and MCC=0.980) performed similarly. The worst results were obtained by the transformer architectures; both ViT (F1=0.409 and MCC=0.0) and Swin Transformer (F1=0.977 and MCC=0.956) obtained the worst results.

In the manuscript, we discussed an approach for training the neural network using digital twin data. The proposed approach has several limitations. It assumes that the quality of the generated data is similar or identical to that of real-world acoustic sonar data, which is not always true. Many elements may cause issues in real data; therefore, models trained on synthetic data should be retrained on real-world data before application. But since the models are already trained, a significantly smaller amount of real data is required. It is also worth mentioning that the prepared FLS images are idealistic and assume a flattened seabed without any obstacles.

The conducted studies are part of a larger project in which we are working on developing a multi-view approach for UXO data classification. Therefore, the feature work comprises combining sonar data with the magnetometry data presented in [19], resulting in a multi-modal view of UXO objects. We assume that this will improve the present prediction performance. In our opinion, it is also worth investigating the idea of using of a series of sonar images representing a single object.

## Figures and Tables

**Table 1:** Generator parameters.

Parameter	Value
Sonar update rate	2 Hz
Sonar horizontal ray samples	512
Sonar horizontal FOV	90°
Sonar vertical ray samples	300
Sonar vertical FOV	20°
Sonar min range	0.1 m
Sonar max range	10 m
Sonar range resolution	0.1 m
Sonar frequency	900 kHz
Sound speed	1500 m/s

**Table 2:** Dataset summary.

Parameter	Sonar Images	Sonar Cases
Total number	69,444	23,148
Number of UXOs	21,318	7106
Number of nonUXOs	48,126	16,042
UXO percentage	30%	
nonUXO percentage	70%	
Train/Validate/Test split	65%/5%/30%	
Train/Validate/Test split for k-fold	72%/8%/20%	

**Table 3:** Resnet50 results.

TL Mode	Learning Rate (LR)	F1 Score (Macro F1)	Average Precision Score (AP)	Balanced Accuracy Score (BA)	Matthews Correlation Coefficient (MCC)
ALL	0.0001	0.986	0.967	0.988	0.973
S3+S4+S5+FC	0.0001	0.983	0.963	0.980	0.966
S4+S5+FC	0.0001	0.984	0.964	0.982	0.968
S5+FC	0.0001	0.971	0.936	0.969	0.942
FC	0.0001	0.813	0.631	0.808	0.626
S1+FC	0.0001	0.924	0.834	0.922	0.847
S1+S2+FC	0.0001	0.986	0.968	0.984	0.971
S5+FC	0.001	0.409	0.307	0.500	0.000
S5+FC	0.0001	0.974	0.941	0.973	0.948
S5+FC	0.00001	0.963	0.917	0.962	0.926

**Table 4:** Model comparison—cross-validation results.

Model	Parameters	F1 Score ((Macro) F1)	Average Precision Score (AP)	Balanced Accuracy Score (BA)	Matthews Correlation Coefficient (MCC)
VGG16	ALL LR = 0.00001	$0.994\pm 5.97\times 10^{-4}$	$0.985\pm 1.75\times 10^{-3}$	$0.994\pm 4.46\times 10^{-4}$	$0.987\pm 1.19\times 10^{-3}$
ResNet34	ALL LR = 0.0001	$0.991\pm 2.17\times 10^{-3}$	$0.980\pm 5.37\times 10^{-3}$	$0.991\pm 1.90\times 10^{-3}$	$0.983\pm 4.34\times 10^{-3}$
ResNet50	ALL LR = 0.0001	$0.990\pm 9.34\times 10^{-4}$	$0.977\pm 2.37\times 10^{-3}$	$0.990\pm 1.17\times 10^{-3}$	$0.980\pm 1.87\times 10^{-3}$
RegNetY800MF	ALL LR = 0.0001	$0.994\pm 4.61\times 10^{-4}$	$0.980\pm 5.81\times 10^{-3}$	$0.992\pm 2.04\times 10^{-3}$	$0.983\pm 4.63\times 10^{-3}$
ViT	ALL LR = 0.0001	0.409 ± 0	0.307 ± 0	0.5 ± 0	0 ± 0
SwinTransformer	ALL LR = 0.0001	$0.977\pm 8.55\times 10^{-3}$	$0.945\pm 1.96\times 10^{-2}$	$0.978\pm 8.27\times 10^{-3}$	$0.956\pm 1.71\times 10^{-2}$

## Data Availability

Data generated using the generator discussed in this paper are available at https://www.kaggle.com/datasets/piotres/front-looking-sonar-uxo.

## Abbreviations

The following abbreviations are used in this manuscript:

CNN	Convolutional neural network
LR	Learning rate
ML	Machine learning
MLO	Mine-like object
TL	Transfer learning
UXO	Unexploded ordnance
